# A high degree of knee flexion after TKA promotes the ability to perform high-flexion activities and patient satisfaction in Asian population

**DOI:** 10.1186/s12891-021-04369-4

**Published:** 2021-06-21

**Authors:** Hyuk-Soo Han, Jong Seop Kim, Bora Lee, Sungho Won, Myung Chul Lee

**Affiliations:** 1 Department of Orthopaedic Surgery, Seoul National University Hospital, 101 Daehak-ro, Jongno-gu, Seoul, 03080 South Korea; 2 Department of Statistics, Graduate School of Chung-Ang University, Seoul, South Korea; 3 Department of Public Health Sciences, Seoul National University, Seoul, South Korea

**Keywords:** Knee flexion, High-flexion activity, Patient satisfaction, Quality of life, Total knee arthroplasty

## Abstract

**Background:**

This study investigated whether achieving a higher degree of knee flexion after TKA promoted the ability to perform high-flexion activities, as well as patient satisfaction and quality of life.

**Methods:**

Clinical data on 912 consecutive primary TKA cases involving a single high-flexion posterior stabilized fixed-bearing prosthesis were retrospectively analyzed. Demographic and clinical data were collected, including knee flexion angle, the ability to perform high-flexion activities, and patient satisfaction and quality of life.

**Results:**

Of the cases, 619 (68%) achieved > 130° of knee flexion after TKA (high flexion group). Knee flexion angle and clinical scores showed significant annual changes, with the maximum improvement seen at 5 years and slight deterioration observed at 10 years postoperatively. In the high flexion group, more than 50% of the patients could not kneel or squat, and 35% could not stand up from on the floor. Multivariate analysis revealed that > 130° of knee flexion, the ability to perform high-flexion activities (sitting cross-legged and standing up from the floor), male gender, and bilateral TKA were significantly associated with patient satisfaction after TKA, while the ability to perform high-flexion activities (sitting cross-legged and standing up from the floor), male gender, and bilateral TKA were significantly associated with patient quality of life after TKA.

**Conclusions:**

High knee flexion angle (> 130°) after TKA increased the ease of high-flexion activities and patient satisfaction. The ease of high-flexion activities also increased quality of life after TKA in our Asian patients, who frequently engage in these activities in daily life.

## Background

The main goals of total knee arthroplasty (TKA) in older patients are pain relief and functional improvement in common activities of daily living (ADL) [1]. Restoration of knee flexion is an important determinant of the functional outcome after TKA. Flexion beyond 110° improves functional ability [2, 3], and patients with a range of motion (ROM) of 128–132° achieved the best functional results [4]. Knee flexion < 130° after TKA precluded the performance of high-flexion activities, such as squatting, sitting cross-legged, or kneeling in Asian populations [5]. Similarly, TKA failed to meet expectation for high-flexion activities in Western populations [6]. Crouching and kneeling are the activities most limited in patients with osteoarthritis of the knee [7]. Following TKA, kneeling was reported as the second most difficult activity to perform, after squatting. Failure to restore the ability to kneel and squat, and the importance of these movements to ADL, may contribute to lower satisfaction with TKA.

High-flexion TKA is designed to achieve the > 130° of knee flexion necessary for ADL, including kneeling and gardening [8]. However, some patients do not achieve satisfactory flexion or performance of high-flexion activities after TKA. Although limited ROM is a significant cause of poor functional outcome and patient dissatisfaction, the associations among high degree of flexion, ability to perform high-flexion activities, and patient satisfaction after TKA are rarely studied [2, 9]. The existing studies involved Western patients, who do not tend to use deep flexion frequently compared to Asian patients.

Therefore, this study investigated whether a higher degree of knee flexion after high-flexion TKA is associated with the performance of high-flexion activities, increased patient satisfaction and higher quality of life in an Asian population. We hypothesized that high flexion (> 130°) of TKA knees postoperatively is associated with enhanced performance of high-flexion activities, and improved patient satisfaction and quality of life.

## Methods

**Fig. 1:**
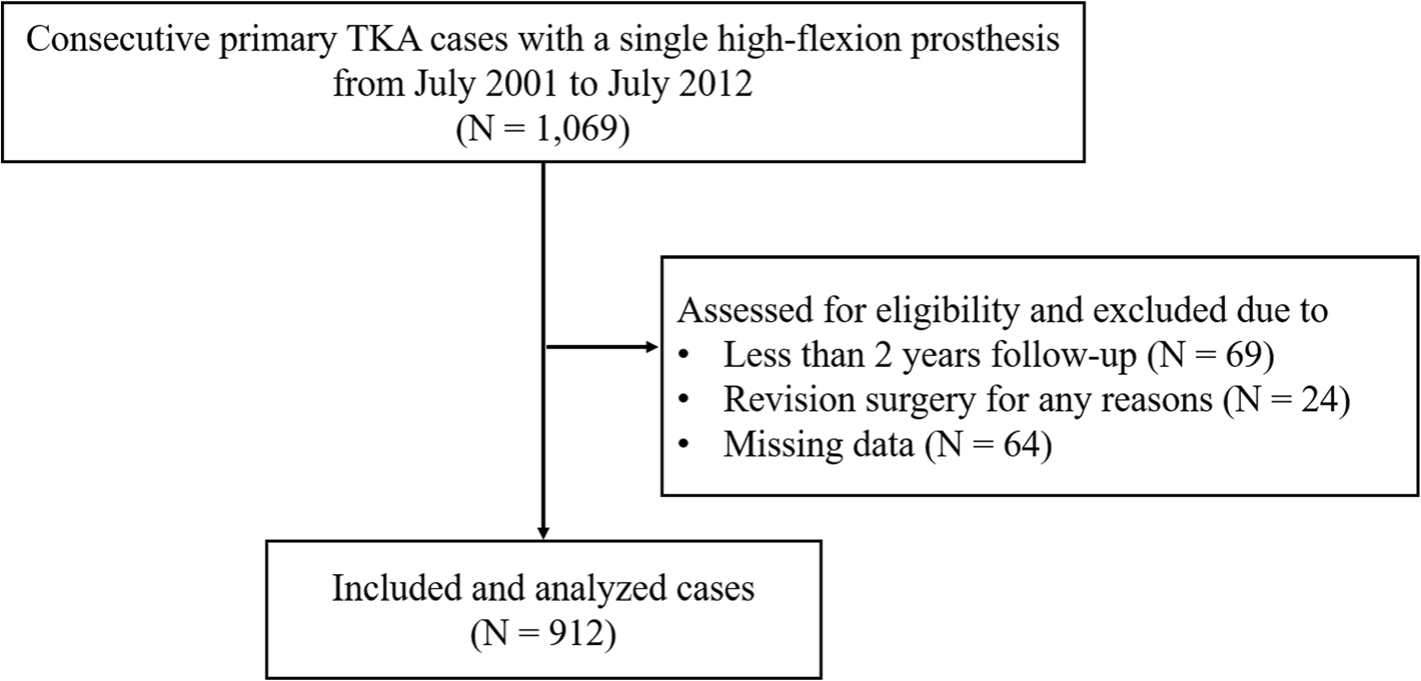
Flow diagram for the patient enrollment

We retrospectively reviewed prospectively collected data for 1069 consecutive primary TKA cases using a single high-flexion posterior stabilized fixed-bearing prosthesis (NexGen®; Zimmer, Warsaw, IN, USA) from July 2001 to July 2012. Of the 1069 knees, 157 were excluded from the analysis because the duration of follow-up was less than 2 years (*n* = 69) or they had revision surgeries (n-24) or missing data (*n* = 64), leaving 912 knees in 610 patients eligible for this study (Fig. 1). There were 43 men and 567 women (mean age, 69 years; range: 41–87 years). The median follow-up was 5.0 years (range: 2.0–14.3 years). The main diagnosis for TKA was osteoarthritis (903 knees; 99%).

The primary TKAs were performed by a single surgeon using conventional instruments. The posterior cruciate ligament was sacrificed and fixed-bearing tibia inserts were implanted in all patients. The patella was selectively resurfaced and the indications for patellar preservation were a thin patella (< 20 mm thick intraoperatively), nearly normal articular cartilage (International Cartilage Repair Society [ICRS] Grade 0 or 1), no preoperative patellar pain, or poor patellar bone quality. All prostheses were fixed with cement. All patients followed the same postoperative rehabilitation protocol, starting continuous passive motion the day after surgery and beginning full weight-bearing (as tolerated) 2 days after surgery. Passive gentle flexion was performed until the patients achieved nearly 130° of knee flexion.

Patients were clinically assessed preoperatively, postoperatively at 6 weeks, 3 months, and 1 year, and annually thereafter using the ROM, Knee Society knee score (KSS), Knee Society function score (KSFS), Hospital for Special Surgery (HSS) score, and Western Ontario and McMaster Universities Osteoarthritis Index (WOMAC). The non-weight-bearing maximal knee flexion angle was measured using a standard goniometer in supine position by two independent physician assistants, preoperatively and at each follow-up. While measuring the maximal knee flexion angle, patients were asked to bend their knees as much as they could or until they felt slight pain while lying in a supine position. To evaluate the ability to perform high-flexion activities, the patients were asked about their ability to kneel, squat, sit cross-legged, and rise after sitting on the floor. The ability to do high-flexion activities was documented by easiness; impossible, hard to do, or easy to do. A questionnaire scored on a 5-point Likert scale (completely satisfied, 5; satisfied, 4; barely acceptable, 3; unsatisfied, 2; completely unsatisfied, 1) was used to evaluate patient satisfaction, and a questionnaire scored on a 7-point Likert scale was used to evaluate the change in patient quality of life [10, 11]. The longitudinal data including preoperative and postoperative 2-, 5-, and 10-year outcome were used in the analysis, except the final knee flexion angle, satisfaction and quality of life in which the most recent follow-up data were used.

This study complied with the Helsinki Declaration and was approved by the ethics committee of Seoul National University Hospital (IRB No. 0603–105-170).

### Statistical analysis

Continuous data are provided as the mean and standard deviation, while categorical data are presented as frequencies and proportions. The consecutive patients were divided into two groups according to whether or not > 130° of knee flexion was achieved. The groups were compared using Student’s *t*-test or Wilcoxon’s rank-sum test for continuous data, according to the normality of the data distribution. Pearson’s chi-square test or Fisher’s exact test was used to compare categorical data depending on whether the assumptions for Pearson’s chi-square test were met. Within group pre- and postoperative continuous data were compared with paired *t*-tests.

Linear mixed models were generated about the ability to perform high-flexion activities with patient as a random effect and assuming a random slope for the follow-up time. Based on these models, estimated marginal least-square means were calculated at four time points: preoperatively, and at 2, 5, and 10 years postoperatively. Group (final knee flexion < 130° vs. ≥ 130°) and follow-up time were included as main effects, in addition to the interaction term and covariates of age, sex, body mass index (BMI), bilateral TKA, and patellar resurfacing. The follow-up time was modeled as a quadratic polynomial. The covariance matrix was selected based on the Akaike information criterion among an unstructured, compound symmetric, or autoregression (1) matrix. Significant differences between the groups were tested for at each time point.

A generalized estimating equation was fitted to the performance of high-flexion activities, patient satisfaction, and quality of life after TKA, considering each patient as a random effect. The group and follow-up time were included as main effects, along with the covariates age, sex, BMI, bilateral TKA, and patellar resurfacing. A cumulative logits (proportional odds) model was used, since the proportional odds assumptions were upheld. As the postoperative outcome, we estimated the probability of having the ability to perform high-flexion activities after 2, 5, and 10 years. Odds ratios were calculated for the performance of high-flexion activities or knee flexion ≥130°, as a measure of patient satisfaction and quality of life.

A two-sided *p* < 0.05 was considered statistically significant and all analyses were performed using R software (ver. 3.6.1; The R Foundation for Statistical Computing, Vienna, Austria).

## Results

Table 1 summarizes the demographic and preoperative characteristics according to final knee flexion after TKA. Knees with ≥130° of flexion after TKA (Group H, *n* = 619, 68%) had a greater preoperative flexion angle than those that had < 130° of flexion after TKA (Group N, *n* = 291, 32%). No other demographic or preoperative clinical variables differed between the two groups. The proportions of the cases with patella resurfacing were similar in the two groups (group H, *n* = 516, 83.4% vs. group N, *n* = 251, 86.3%). Table 2 summarizes the mean annual changes in clinical variables in both groups after TKA. Knee flexion angle, KSFS, HSS knee score, and WOMAC total and physical function scores after TKA showed significant annual changes in both groups. These clinical variables showed maximal improvement at 5 years postoperatively, and had deteriorated slightly at 10 years postoperatively. Except for knee flexion angle, the clinical variables did not differ significantly between the two groups. Table 3 summarizes the ability to perform high-flexion activities after TKA in both groups. A larger proportion of patients in Group H could easily perform various high-flexion activities compared with Group N (*p* < 0.001). The proportions of patients who could kneel and squat easily showed a trend to increase with time in both groups, although the changes showed no statistical significances. However, the proportions of those who could sit cross-legged or stand up from the floor easily did not change over time.

**Table 1:** The demographics and preoperative characteristics according to final knee flexion after TKA

Variable	Final knee flexion		***p***-value
	≥ 130° (***N*** = 619)	< 130° (***N*** = 291)	
Age (years)	69.1 ± 6.6	68.4 ± 8.4	n.s.
Sex (female)	575 (92.9%)	274 (94.2%)	n.s.
Height (cm)	152.9 ± 6.3	152.3 ± 6.7	n.s.
Weight (kg)	62.5 ± 8.6	62.9 ± 8.9	n.s.
Body mass index (kg/m^2^)	26.7 ± 3.1	27.1 ± 3.4	n.s.
Diagnosis			n.s.
Osteoarthritis	613 (99.0%)	288 (99.0%)	
Rheumatoid arthritis	2 (0.3%)	2 (0.7%)	
Others	4 (0.6%)	1 (0.3%)	
Side (right)	306 (49.4%)	154 (52.9%)	n.s.
Bilateral TKA	426 (68.8%)	183 (62.9%)	n.s.
Patellar resurfacing	516 (83.4%)	251 (86.3%)	n.s.
Additional lateral release	4 (0.7%)	3 (1.0%)	n.s.
History of operation on the knee	11 (1.8%)	10 (3.4%)	n.s.
Follow-up time (years)	5.1 [2.9, 7.8]	4.6 [2.0, 7.0]	0.010
Preoperative functional evaluation			
Knee flexion (°)	129.2 ± 11.4	113.9 ± 19.8	< 0.001
Flexion contracture (°)	12.3 ± 8.2	13.8 ± 8.6	0.012
Range of motion (°)	116.8 ± 15.4	100.1 ± 23.3	< 0.001
Knee Society knee score	47.4 ± 16.8	46.1 ± 18.2	n.s.
Knee Society function score	41.3 ± 18.9	42.0 ± 19.3	n.s.
Hospital for Special Surgery score	56.6 ± 15.2	55.0 ± 15.6	n.s.
WOMAC - pain	9.1 ± 3.5	8.9 ± 3.4	n.s.
WOMAC - stiffness	4.2 ± 1.9	4.1 ± 1.9	n.s.
WOMAC - physical function	38.2 ± 14.6	37.9 ± 14.0	n.s.
WOMAC total	50.4 ± 18.8	49.7 ± 18.3	n.s.

*WOMAC* Western Ontario and McMaster Universities Osteoarthritis Index, *n.s*. not significant

Data was reported as mean ± standard deviation or median [interquartile range (IQR)] for continuous variable and frequency (percentage) for categorical variables

*P*-values were calculated by Student’s t-test or Mann-Whitney U test for continuous variables and chi-square test or Fisher’s exact test for categorical variables as appropriate

**Table 2:** Mean annual change of clinical variables in groups according to final knee flexion after TKA

Variable	Estimated marginal mean (95% CI)				Mean change/year (95% CI)		p for interaction with time		
	Preoperative (***n*** = 912)	2 years (***n*** = 912)	5 years (***n*** = 718)	10 year (***n*** = 261)	linear	quadratic	linear		quadratic
Knee flexion (°)									
Final knee flexion < 130	119.5 (114.6, 124.3)	123.0 (118.2, 127.8)	124.7 (119.8, 129.6)	118.2 (112.5, 123.8)	2.2 (1.6, 2.9)	−0.2 (− 0.3, − 0.2)	0.006		0.023
Final knee flexion ≥130	131.4 (126.6, 136.2)***	134.6 (129.8, 139.3)***	137.3 (132.5, 142.1)***	136.6 (131.5, 141.7)***	1.9 (1.4, 2.3)	−0.1 (− 0.2, − 0.1)			
Flexion contracture (°)									
Final knee flexion < 130	10.4 (8.2, 12.5)	3.3 (1.2, 5.4)	−2.1 (−4.2, 0.0)	2.8 (0.4, 5.2)	−4.2 (− 4.5, −3.9)	0.4 (0.3, 0.4)	n.s.		n.s.
Final knee flexion ≥130	9.7 (7.6, 11.7)	3.2 (1.1, 5.2)	−1.9 (−4.0, 0.2)	2.0 (−0.2, 4.2)	−3.9 (− 4.1, − 3.7)	0.3 (0.3, 0.3)			
Range of motion (°)									
Final knee flexion < 130	107.7 (100.2, 115.2)	117.4 (109.9, 124.8)	124.0 (116.5, 131.6)	114.3 (105.6, 123.1)	5.9 (4.8, 7.0)	−0.5 (− 0.7, − 0.4)	n.s.		0.028
Final knee flexion ≥130	119.5 (112.1, 126.9)	127.7 (120.4, 135.1)	134.8 (127.4, 142.2)	132.5 (124.6, 140.4)**	4.8 (4.1, 5.5)	−0.4 (− 0.4, − 0.3)			
Knee Society score									
Final knee flexion < 130	58.9 (52.4, 65.5)	85.7 (79.3, 92.1)	105.9 (99.4, 112.4)	86.2 (78.5, 94.0)	16.1 (15.0, 17.1)	−1.3 (− 1.5, − 1.2)	n.s.		n.s.
Final knee flexion ≥130	59.2 (52.7, 65.6)	85.9 (79.6, 92.3)	106.2 (99.8, 112.6)	86.9 (80.1, 93.8)	16.0 (15.3, 16.7)	−1.3 (− 1.4, − 1.3)			
Knee Society function score									
Final knee flexion < 130	50.3 (40.3, 60.3)	73.0 (63.2, 82.9)	87.2 (77.2, 97.3)	57.7 (45.9, 69.5)	14.0 (12.7, 15.4)	−1.3 (− 1.5, − 1.2)	0.007		n.s.
Final knee flexion ≥130	50.7 (40.8, 60.6)	73.5 (63.6, 83.3)	90.1 (80.2, 100.0)	71.0 (60.4, 81.5)	13.7 (12.9, 14.6)	−1.2 (− 1.3, − 1.1)			
Hospital for Special Surgery score									
Final knee flexion < 130	63.7 (57.5, 69.9)	83.8 (77.6, 89.9)	97.9 (91.7, 104.1)	78.8 (71.5, 86.0)	12.2 (11.4, 13.0)	−1.1 (− 1.2, − 1.0)	0.029		n.s.
Final knee flexion ≥130	64.1 (58.0, 70.2)	84.7 (78.7, 90.8)	100.2 (94.1, 106.3)	84.8 (78.3, 91.3)	12.4 (11.8, 12.9)	−1.0 (− 1.1, − 1.0)			
WOMAC (total)									
Final knee flexion < 130	39.9 (32.6, 47.1)	19.9 (12.7, 27.0)	4.9 (−2.4, 12.1)	20.0 (11.9, 28.1)	−12.0 (− 13.0, − 11.1)	1.0 (0.9, 1.1)	0.003		n.s.
Final knee flexion ≥130	40.7 (33.5, 47.8)	18.8 (11.7, 26.0)	1.8 (−5.4, 8.9)	15.0 (7.5, 22.5)	−13.0 (− 13.6, − 12.4)	1.0 (1.0, 1.1)			
WOMAC (pain)									
Final knee flexion < 130	6.4 (5.1, 7.6)	2.1 (0.9, 3.4)	−1.3 (−2.5, −0.0)	1.0 (−0.4, 2.4)	− 2.5 (− 2.7, − 2.3)	0.2 (0.2, 0.2)	n.s.		n.s.
Final knee flexion ≥130	6.6 (5.4, 7.8)	2.1 (0.9, 3.3)	− 1.5 (− 2.7, − 0.3)	1.1 (− 0.2, 2.4)	−2.7 (− 2.8, − 2.6)	0.2 (0.2, 0.2)			
WOMAC (stiffness)									
Final knee flexion < 130	3.2 (2.5, 3.9)	1.4 (0.7, 2.1)	0.0 (− 0.7, 0.7)	1.0 (0.2, 1.8)	−1.1 (− 1.2, − 1.0)	0.1 (0.1, 0.1)	n.s.		0.027
Final knee flexion ≥130	3.3 (2.6, 4.1)	1.3 (0.6, 2.0)	−0.2 (− 0.9, 0.5)	1.1 (0.4, 1.8)	−1.2 (− 1.3, − 1.1)	0.1 (0.1, 0.1)			
WOMAC (physical function)									
Final knee flexion < 130	30.8 (24.9, 36.6)	16.2 (10.5, 21.9)	5.7 (−0.1, 11.5)	18.6 (12.0, 25.2)	−8.8 (−9.6, −8.0)	0.76 (0.67, 0.85)	0.001		n.s.
Final knee flexion ≥130	31.0 (25.3, 36.7)	15.2 (9.5, 20.9)	3.0 (−2.7, 8.7)	13.3 (7.3, 19.3)	−9.4 (−9.9, −8.9)	0.77 (0.71, 0.82)			

*WOMAC* Western Ontario and McMaster Universities Osteoarthritis Index, *n.s*. not significant

***, *p* < 0.001; **, *p* < 0.01; *, *p* < 0.05, compared two group at each point

**Table 3:** Ability to do high-flexion activities in groups according to final knee flexion after TKA at follow-up

High-flexion activity	Impossible (Estimated proportion, %)^**a**^			Hard to do (Estimated proportion, %)^**a**^			Easy to do (Estimated proportion, %)^**a**^			Odd ratio for easy to do (95% CI)	***p***-value	p for interaction with time
	2 years	5 years	10 years	2 years	5 years	10 years	2 years	5 years	10 years			
Kneel												
Final knee flexion < 130°	80.0	68.8	45.1	14.1	21.0	31.6	5.9	10.2	23.3	1 (Reference)	< 0.001	n.s.
Final knee flexion ≥130°	59.2	50.2	35.5	26.1	29.9	33.3	14.7	19.9	31.3	3.25 (1.88–5.64)		
Squat												
Final knee flexion < 130°	72.9	60.5	37.5	18.6	25.5	33.1	8.5	14.0	29.4	1 (Reference)	< 0.001	n.s.
Final knee flexion ≥130°	47.1	39.4	27.8	31.0	32.8	32.8	22.0	27.8	39.4	3.56 (2.08–6.12)		
Sit cross-legged												
Final knee flexion < 130°	41.5	36.5	28.7	32.4	33.2	33.0	26.0	30.3	38.3	1 (Reference)	< 0.001	n.s.
Final knee flexion ≥130°	13.9	13.9	13.9	25.4	25.3	25.3	60.7	60.8	60.9	5.04 (2.95–8.63)		
Stand from the floor												
Final knee flexion < 130°	14.5	15.1	16.1	59.5	58.4	56.6	25.9	26.5	27.3	1 (Reference)	< 0.001	n.s.
Final knee flexion ≥130°	35.8	29.5	20.6	31.0	37.4	49.0	33.3	33.1	30.4	4.09 (2.21–7.58)		

*n.s.* not significant

^a^ Estimated after adjustment for age, sex, body mass index, bilateral TKA, and patellar resurfacing

Tables 4 and 5 summarize the results of uni- and multivariate analyses of the association of > 130° of knee flexion with patient satisfaction and quality of life after TKA. Postoperative knee flexion angle, > 130° of knee flexion, ability to perform high-flexion activities (sit cross-legged and stand up from the floor), male gender, and bilateral TKA were significant perioperative predictors of patient satisfaction in the univariate analyses. Multivariate analysis revealed that > 130° of knee flexion, ability to perform high-flexion activities (sit cross-legged and stand up from the floor), male gender, and bilateral TKA remained as factors significantly associated with patient satisfaction after TKA. Similarly, the postoperative knee flexion angle, > 130° of knee flexion, ability to perform high-flexion activities (sit cross-legged and stand up from the floor), male gender, and bilateral TKA were significant perioperative predictors of quality of life in univariate analyses; the ability to perform high-flexion activities (sit cross-legged and stand up from the floor), male gender, and bilateral TKA remained significant in multivariate analysis.

**Table 4:** Multivariable analysis results for the association between more than 130 degrees of knee flexion and patients’ satisfaction after TKA

Variable		Univariable		Multivariable		Multivariable	
		OR (95% CI)	***p***-value	OR (95% CI)	***p***-value	OR (95% CI)	***p***-value
Knee flexion after TKA (°)		1.02 (1.01–1.03)	0.001	1.008 (0.997–1.019)	n.s.		
≥130° of Knee flexion after TKA (°)							
No		1 (Reference)				1 (Reference)	
Yes		1.82 (1.34–2.47)	< 0.001			1.38 (1.007–1.889)	0.045
Ability to do high-flexion activities							
Kneel (reference: impossible)	Hard to do	0.78 (0.57–1.06)	n.s.				
	Easy to do	1.07 (0.61–1.88)	n.s.				
Squat (reference: impossible)	Hard to do	0.76 (0.55–1.03)	n.s.				
	Easy to do	0.95 (0.55–1.65)	n.s.				
Sit cross-legged (reference: impossible)	Hard to do	1.71 (1.23–2.39)	0.001	1.426 (1.015–2.003)	0.041	1.414 (1.008–1.985)	0.045
	Easy to do	3.89 (2.58–5.87)	< 0.001	2.557 (1.64–3.988)	< 0.001	2.458 (1.573–3.842)	< 0.001
Stand from the floor (reference: impossible)	Hard to do	3.89 (1.81–8.34)	< 0.001	3.104 (1.403–6.87)	0.005	3.107 (1.405–6.867)	0.005
	Easy to do	8.15 (3.63–18.28)	< 0.001	4.429 (1.886–10.4)	0.001	4.45 (1.897–10.438)	0.001
Age (years) (reference: < 60)	60–74	0.86 (0.5–1.48)	n.s.				
	≥ 75	0.69 (0.36–1.31)	n.s.				
Male (vs. female)		2.51 (1.32–4.8)	0.005	2.282 (1.217–4.278)	0.01	2.312 (1.234–4.331)	0.009
Body mass index (kg/m^2^) (reference: < 25)	25 - < 30	1.27 (0.88–1.84)	n.s.				
	≥ 30	1.07 (0.66–1.73)	n.s.				
Bilateral TKA (vs. unilateral TKA)		1.41 (1.01–1.98)	0.046	1.443 (1.035–2.013)	0.031	1.456 (1.045–2.028)	0.026
Patellar resurfacing (vs. un-resurfacing)		0.68 (0.45–1.04)	n.s.				

*OR* odds ratio, *CI* confidence interval, *n.s.* not significant

**Table 5:** Multivariable analysis results for the association between more than 130 degrees of knee flexion and patients’ quality of life after TKA

Variable		Univariable		Multivariable		Multivariable	
		OR (95% CI)	***p***-value	OR (95% CI)	***p***-value	OR (95% CI)	***p***-value
Knee flexion after TKA (°)		1.01 (1–1.02)	0.025	1.003 (0.993–1.013)	0.569		
≥130° of Knee flexion after TKA (°)							
No		1 (Reference)				1 (Reference)	
Yes		1.5 (1.12–2)	0.006			1.17 (0.872–1.569)	n.s.
Ability to do high-flexion activities							
Kneel (reference: impossible)	Hard to do	0.96 (0.72–1.27)	n.s.				
	Easy to do	0.94 (0.55–1.62)	n.s.				
Squat (reference: impossible)	Hard to do	0.86 (0.65–1.14)	n.s.				
	Easy to do	1.09 (0.65–1.82)	n.s.				
Sit cross-legged (reference: impossible)	Hard to do	1.25 (0.92–1.7)	n.s.	1.039 (0.755–1.428)	n.s.	1.028 (0.748–1.413)	n.s.
	Easy to do	2.97 (2.08–4.24)	< 0.001	1.787 (1.204–2.653)	0.004	1.74 (1.169–2.589)	0.006
Stand from the floor (reference: impossible)	Hard to do	3.08 (1.38–6.87)	0.006	2.639 (1.172–5.942)	0.019	2.619 (1.163–5.898)	0.02
	Easy to do	8.25 (3.59–18.98)	< 0.001	5.359 (2.267–12.668)	< 0.001	5.325 (2.252–12.589)	< 0.001
Age (years) (reference: < 60)	60 - < 75	1.25 (0.76–2.04)	0.38				
	≥ 75	1.14 (0.64–2.04)	n.s.				
Male (vs. female)		2.28 (1.31–3.97)	0.004	2.379 (1.383–4.092)	0.002	2.388 (1.388–4.11)	0.002
Body mass index (kg/m^2^) (reference: < 25)	25 - < 30	1.21 (0.87–1.7)	n.s.				
	≥ 30	1.11 (0.71–1.74)	n.s.				
Bilateral TKA (vs. unilateral TKA)		1.57 (1.15–2.15)	0.005	1.66 (1.22–2.259)	0.001	1.663 (1.222–2.262)	0.001
Patellar resurfacing (vs. un-resurfacing)		0.87 (0.6–1.27)	n.s.				

*OR* odds ratio, *CI* confidence interval, *n.s.* not significant

## Discussion

The most important findings of this study were that two-thirds (619/912, 67.9%) of Asian osteoarthritis patients could achieve high flexion (> 130°) after TKA, which would increase the ease of high-flexion activities (sitting cross-legged and standing up from the floor) and patient satisfaction. The performance of high-flexion activities also increased the quality of life after TKA, while postoperative high flexion of TKA knees did not.

Greater flexion is believed to improve the clinical outcomes of TKA [9]. However, the relationship between ROM and functional outcome is unclear. Some studies have reported that greater flexion after TKA is correlated with improved clinical outcomes and quality of life [12, 13], whereas another found no correlation between greater flexion and clinical outcomes [3]. Most of these studies evaluated Western patients who had an average knee flexion < 120° [14]. Moreover, most patient-based questionnaires were not designed for use in high-flexion TKA patients (e.g., no extra points were scored for ROM > 125°). Therefore, data on whether greater knee flexion leads to improved patient satisfaction and quality of life after TKA remain limited. In this study, we compared patient satisfaction and quality of life after TKA between groups who did and did not achieve > 130° of knee flexion. The ability to perform several high-flexion activities was also evaluated, to investigate the relationship with patient satisfaction and quality of life.

Despite the overall favorable results after TKA, studies have estimated that 11–20% of TKA patients are dissatisfied after surgery [7, 15, 16]. However, significant differences in satisfaction rates and the kinds of limited activities after TKA are seen between Western and Asian populations [17–19], which might arise from differences in patient expectations and living habits. Most ADLs require 90–120° knee flexion, while kneeling, squatting, and sitting cross-legged, which necessitate flexion of the knee joint beyond 120°, are also required for various lifestyle activities, including cultural and religious activities in Asian populations [20]. In one study, TKA failed to meet expectations regarding kneeling, squatting, and stair climbing [6]. In a prospective cohort, the largest proportions of patients with unfulfilled expectations were those unable to kneel (47%) or squat (44%) [21]. To meet patient expectations and ensure satisfaction, it is important to reproduce the pre-arthritic knee flexion angle after TKA [9]. However, high-flexion activities are also affected by the efficiency of the quadriceps, stability, and kinematics during deep knee flexion [22, 23]. In a retrospective study of 1013 TKAs of 748 Chinese patients, the top six items with respect to dissatisfaction were sitting with the legs crossed, squatting, walking fast or jogging, knee clunking, abnormal feeling in the knee, and climbing stairs [17]. More than half of the patients in their study were not satisfied with their ability to squat. In another survey of an Asian population, high-flexion activity ranked lowest for satisfaction among the study variables, and was one of the highest ranked variables in which improvement was desired, reflecting its importance to patients after TKA [24]. Although we included more than 600 knees with > 130° of knee flexion after TKA, the ability to kneel or squat was not achieved in more than 50% of the patients, and the ability to stand from the floor was not achieved in more than 35%. This poor rate of kneeling and squatting ability is consistent with other studies [6, 21, 25], although those studies did not investigate patient satisfaction. However, high flexion is not always reported to be correlated with functional outcome. A retrospective review of TKAs performed due to a diagnosis of osteoarthritis reported that obtaining deep flexion conferred no benefit regarding overall knee function [3]. Another study reported no significant difference in satisfaction among three groups classified according to knee flexion: low (≤ 110°), intermediate (111–130°), or high (> 130°) [2]. Two other studies similarly found no significant correlation of flexion with patient satisfaction or pain, although there was a positive correlation between increased postoperative flexion and the ability to perform ADL [4, 12]. However, those studies included relatively few cases, and most examined Western populations, in which the average knee flexion is typically low.

Several other factors have been suggested to influence patient satisfaction, including the diagnosis, deformity, age, gender, surgical technique, postoperative pain control and rehabilitation, and lifestyle [26]. In the present study, male gender and bilateral TKA were significantly associated with patient satisfaction and quality of life after TKA. In a previous study of the factors predicting the Forgotten Joint Score after TKA, the “excellent” cluster included mainly male patients with high flexion and low BMI [27]. Another study reported that bilateral TKA was found to be more common in the satisfied group (77.8%) than in the dissatisfied group (66.3%), although the difference was not statistically significant [28].

The surgeon should be aware of the potential complications associated with performing high-flexion activities after TKA, including excessive wear, fracture, and dislocation of the cam-post mechanism. In a previous study, the mean internal rotation of the tibial component during kneeling exceeded the manufacturer’s safety range, increasing the risk of edge loading not only in the posterolateral area of the polyethylene insert, but also in the post-cam contact area [29]. Another study revealed that post-cam contact stress doubled at 150° of knee flexion; as the average internal rotation of the tibia was > 10°, at which point edge loading readily occurs in this type of prosthesis [30]. Deep-flexion activities generate 1- to 13-times higher net quadriceps moments than walking. High flexion may also be associated with TKA cam-post instability. An in vivo study reported greater contact stress with increasing flexion, which could potentially lead to greater wear, increased patellar fracture, or loosening and earlier failure of the polyethylene insert [31]. They also observed cam-post disengagement at high flexion angles.

Our study was a retrospective review of a prospectively collected database and had several limitations. First, this study was not a prospective controlled one. We grouped the cases according to the postoperative knee flexion angle without matching related factors, which might cause insufficient statistical power. Second, we focused on clinical outcome including high flexion activities, patient satisfaction and quality of life. Radiological outcomes and implant survival were not analyzed. Third, most of the enrolled patients had a diagnosis of osteoarthritis and were female. However, female predominance is a feature of Asian populations undergoing TKA. Our study was also performed in one center, so the influence of cultural and demographic factors on satisfaction and quality of life could not be considered, thus limiting the generalizability. Fourth, we did not evaluate the ability for our patients to perform the high flexion activities preoperatively and patient expectations, where patient satisfaction is closely related to their expectations. Lastly, due to the complexity of our data on different time points, we could not analyze the relationship with patient satisfaction by time point, and we had to analyze it based on recent data. However, despite these partly unavoidable limitations, this study provides detailed insight into the long-term results of TKA.

## Conclusion

The achievement of high flexion (> 130°) after TKA increased the ease of high-flexion activities (sitting cross-legged and standing up from the floor) and patient satisfaction. The ease of high-flexion activities also increased the quality of life after TKA in our Asian population, where such populations frequently engage in these activities during daily life. However, high knee flexion angle after TKA itself did not affect the patients’ quality of life.

## Data Availability

The datasets used and/or analyzed during the current study are available from the corresponding author on reasonable request.

## Abbreviations

TKA: Total knee arthroplasty
ADL: Activities of daily living
ROM: Range of motion
KSS: Knee Society knee score
KSFS: Knee Society function score
HSS: Hospital for Special Surgery
WOMAC: Western Ontario and McMaster Universities Osteoarthritis Index
BMI: Body mass index
